# Heterogeneity and molecular typing characteristics of mast cells in rheumatoid arthritis

**DOI:** 10.3389/fmolb.2026.1898923

**Published:** 2026-07-28

**Authors:** Wenlong Fang, Luyu Jin, Huimin Yu, Yingkun Nie

**Affiliations:** 1 Department of Traditional Chinese Medicine, The Second Affiliated Hospital of Harbin Medical University, Harbin, China; 2 Department of Rheumatology and Immunology, The Second Affiliated Hospital of Harbin Medical University, Harbin, China; 3 Department of Traditional Chinese Medicine, The Second Affiliated Hospital of Zhejiang University, Hangzhou, China

**Keywords:** cellular heterogeneity, immune microenvironment, mast cells, rheumatoid arthritis, single-cell RNA sequencing

## Abstract

**Background:**

Rheumatoid arthritis (RA) is a chronic autoimmune disease characterized by persistent synovial inflammation, immune dysregulation, and progressive joint destruction. Mast cells (MCs) are important components of the synovial immune microenvironment, but their heterogeneity and regulatory roles in RA remain unclear.

**Methods:**

We analyzed single-cell RNA-sequencing data from 17 RA synovial samples in our institutional cohort to construct a cellular atlas of the RA synovial microenvironment. MCs within the myeloid compartment were re-clustered to characterize their heterogeneity, followed by differential expression, functional enrichment, cell-cell communication, and transcriptional regulatory analyses. In parallel, bulk RNA-sequencing data from 218 RA synovial samples in our institutional cohort were analyzed. Consensus clustering based on MC-related signature genes was used to identify molecular subtypes, and pathway activity, immune infiltration, hub genes, and diagnostic performance were further evaluated.

**Results:**

Eleven major cell populations were identified in the single-cell dataset. MCs exhibited distinct transcriptional features and were classified into three heterogeneous subsets associated with protein processing and RNA metabolism, complement and inflammatory regulation, and TNF/NF-κB signaling, respectively. Cell-cell communication analysis revealed extensive interactions between MC subsets and macrophages, monocytes, and cDC2 cells. In the bulk transcriptomic cohort, three stable MC-related molecular subtypes were identified. C2 Subtype showed the strongest inflammatory activation and immune infiltration. Notably, overall MC abundance did not differ significantly among risk groups, suggesting that MC state-associated molecular features, rather than MC quantity, may better distinguish RA subtypes. CCNB1, NCAPH, and DLGAP5 were identified as key hub genes, and an MC score-based diagnostic model showed favorable discriminatory performance.

**Conclusion:**

This study reveals substantial MC heterogeneity in the RA synovial microenvironment and suggests that distinct MC subsets may participate in inflammatory regulation and immune-cell crosstalk. MC-related molecular stratification effectively identifies RA subtypes with different inflammatory phenotypes, supporting the potential value of MC-associated signatures as biomarkers for patient stratification and targeted therapeutic exploration.

## Introduction

Rheumatoid arthritis (RA) is a common chronic autoimmune disease characterized by persistent synovitis, progressive joint destruction, and systemic inflammatory responses, affecting approximately 0.5%–1% of the global population (England et al., 2018; Yip and Navarro-Millan, 2021; Carle et al., 2023). RA typically presents as symmetric polyarthritis, predominantly involving the small joints of the hands and feet, although large joints and extra-articular organs may also be affected. The disease markedly impairs patients’ quality of life and is associated with increased risks of disability and mortality (Cush, 2022). Although considerable progress has been made in the early diagnosis and treatment of RA in recent years, its pathogenesis remains incompletely understood, and curative therapeutic strategies are still lacking in clinical practice. Therefore, further elucidation of the immunopathogenic mechanisms of RA and identification of novel diagnostic biomarkers and therapeutic targets remain of great importance. Recent transcriptomic and bioinformatic studies have further highlighted the value of integrated computational approaches for identifying RA-related molecular mechanisms, immune features, diagnostic biomarkers, and potential therapeutic targets. For example, cuproptosis-related immune characteristic biomarkers and therapeutic targets in RA have been explored through integrated bioinformatics and single-cell RNA-seq analyses, while ferroptosis-related genes and candidate therapeutic drugs have also been identified using bioinformatics-based strategies (Li et al., 2025; Li et al., 2023). These studies provide an important methodological and biological foundation for further dissecting RA heterogeneity from specific immune-cell and molecular-signature perspectives.

The pathological basis of RA mainly includes chronic synovial inflammation, aberrant immune cell infiltration, autoantibody production, and pannus formation (Pappas et al., 2011). Large numbers of abnormally activated immune cells continuously infiltrate the affected joints, forming a complex inflammatory regulatory network that further promotes cartilage and bone erosion and destruction (Schonfeld et al., 2022). Among these, T cells, macrophages, dendritic cells, and B cells play central roles in the initiation and progression of RA (Kondo et al., 2018). T cells can be recruited to the synovium through blood and lymphatic circulation and interact with dendritic cells, macrophages, and B cells to initiate and amplify local inflammatory responses. CD4^+^ T cells are major inflammatory effectors involved in synovial tissue invasion, whereas CD8^+^ T cells contribute to ectopic germinal center formation and exacerbate inflammatory responses (Inamo et al., 2021; Malmstrom et al., 2005). However, beyond these classical lymphoid and myeloid populations, the roles of other immune cell subsets in the RA immune microenvironment, particularly mast cells (MCs), remain insufficiently understood.

MCs are bone marrow-derived innate immune cells that are widely distributed in the skin, respiratory tract, gastrointestinal tract, and connective tissues, where they play important roles in host defense, allergic reactions, inflammatory regulation, and tissue repair (Ribatti and d'Amati, 2023; Jackson et al., 2021). Their cytoplasm is enriched with granules containing a variety of bioactive mediators, including histamine, cytokines, chemokines, proteases, and lipid mediators, enabling them to regulate immune cell recruitment, vascular permeability, inflammatory amplification, and tissue remodeling (Mat et al., 2021). In recent years, the regulatory roles of MCs in a variety of inflammatory diseases and tumor microenvironments have attracted increasing attention. Through interactions with T cells, B cells, macrophages, and other immune cells, MCs participate in maintaining local immune homeostasis and mediating pathological inflammatory responses (Pahima and Dwyer, 2025).

In RA, MCs may represent an important component of the synovial inflammatory microenvironment. Given the persistent inflammatory infiltration, dysregulated cytokine networks, and marked tissue remodeling in RA synovium, mediators released by MCs may contribute to the amplification of synovial inflammation, recruitment of immune cells, and pannus formation, thereby further aggravating cartilage and bone destruction. In addition, MCs can interact with T cells, macrophages, and B cells through the secretion of chemokines and cytokines, thereby influencing the establishment and maintenance of the local immune microenvironment in RA (Lin et al., 2023). Notably, the role of MCs in disease may be dualistic. On one hand, their pro-inflammatory mediators may drive inflammation progression and tissue injury (Hamey et al., 2021); on the other hand, under certain conditions, they may also exert protective effects by modulating immune responses and promoting tissue repair (Lyons and Yi, 2021). Therefore, the precise functions of MCs and their underlying molecular mechanisms in RA remain to be further elucidated.

Overall, the development and progression of RA result from the combined effects of genetic susceptibility, environmental factors, and immune dysregulation. As potentially key immunoregulatory cells in the synovial microenvironment, MCs may participate in multiple pathological processes, including inflammatory amplification, immune cell crosstalk, and joint structural damage. A deeper understanding of the biological functions and molecular mechanisms of MCs in RA will not only advance our knowledge of RA pathogenesis but may also provide new theoretical foundations and potential targets for early diagnosis and precision therapy.

## Materials and methods

### Acquisition and preprocessing of single-cell transcriptomic data

We conducted an analysis of single-cell RNA sequencing (scRNA-seq) data derived from tumor samples of 17 RA synovial tissue samples from patients treated at the Second Affiliated Hospital of Harbin Medical University and included a total of 104,806 cells (HMU cohort, Table 1). The scRNA-seq datasets were processed and converted into Seurat objects using the Seurat R package. Quality control was performed according to the following criteria: only genes expressed in at least five cells were retained; cells expressing fewer than 100 genes were excluded; and cells with more than 5% mitochondrial gene expression were removed. The data were then normalized using the NormalizeData function, and the top 2,000 highly variable genes were identified using the FindVariableFeatures function. Principal component analysis (PCA) was subsequently performed using the RunPCA function, and the first 20 principal components were selected for clustering analysis. Differentially expressed genes (DEGs) for each cluster were identified using the FindAllMarkers function, with thresholds set at false discovery rate (FDR) < 0.05 and |log2 (fold change)| > 0.25.

**TABLE 1:** Rheumatoid arthritis-related dataset.

Cohorts	Samples	Platform	Type
HMU cohort	218	Illumina	RNA-seq
HMU cohort	17	Illumina	scRNA-seq
GSE55235	20	GPL96	Array
GSE55457	23	GPL96	Array
GSE77298	23	GPL570	Array
GSE55584	20	GPL96	Array

Major cell types identified in the single-cell dataset were annotated based on established marker genes, including CD3D, CD8A, CD4, CD56, FOXP3, CD79A, MS4A1, CD14, CD68, COL1A2, COL3A1, VWF, PECAM1, TPSAB1, and CPA3, which were used to define T cells, NK cells, regulatory T cells (Treg cells), B cells, myeloid cells, fibroblasts, endothelial cells, and MCs, respectively.

### Bulk RNA isolation and sequencing

This study included 218 RA synovial tissue samples from patients treated at the Second Affiliated Hospital of Harbin Medical University (HMU cohort, Table 1). The RNA quality and quantity were assessed through several steps: (1) Initial contamination and degradation checks were performed using 1% agarose gel electrophoresis; (2) RNA purity and concentration were measured with a NanoPhotometer® spectrophotometer; (3) RNA integrity was further analyzed using the RNA Nano 6000 Assay Kit on the Bioanalyzer 2100 system. These RNA samples were then used for library preparation. First, mRNA was captured using mRNA Capture Beads with Oligo (dT), followed by purification with Binding and Washing Buffers. The mRNA was then randomly fragmented to 100–200 nt using Fragmentation Buffer and reverse-transcribed into cDNA. The resulting cDNA was purified using DNA Clean Beads and prepared for adaptor ligation. After ligation with adaptors containing UMIs, cDNA underwent one-step PCR amplification. The PCR products were then purified again using DNA Clean Beads and eluted in nuclease-free water. The final library was pooled and sequenced on the Illumina Novaseq 6000 platform, generating 6G of raw paired-end data (150 nt reads).

For initial processing, Perl scripts were used to process raw FASTQ data. Low-quality reads, those containing adapters, or poly-N sequences were removed, resulting in clean reads. All subsequent analyses were performed using these high-quality clean reads. The reference genome (*Homo sapiens* GRCh38.103) index was created using STAR (version 2.7.11b), and the clean reads were aligned to this reference genome with STAR. Gene expression counts were obtained using RSEM (version 1.3.3).

### Acquisition and preprocessing of bulk transcriptomic data

A comprehensive search of the Gene Expression Omnibus (GEO) database was conducted to identify RA-related transcriptomic datasets (Table 1). A total of four microarray datasets comprising 86 RA samples were included in this study (Woetzel et al., 2014; Broeren et al., 2016). The CEL files of the microarray datasets were processed and normalized using the affy R package, and probe IDs were converted into corresponding gene symbols.

### Cell-cell interaction analysis

Cell-cell interaction networks within the RA immune microenvironment were constructed using the Python package CellPhoneDB (v2.0), largely following the default settings of the software (Troule et al., 2025). Potential interaction strength between 2 cell subsets was predicted based on the expression levels of ligand-receptor pairs. Interactions were subsequently filtered according to statistical significance (P < 0.05). Specifically, if a cell expressed a receptor or ligand, the corresponding interaction was defined as an input or output signal, respectively. Biologically relevant ligand-receptor pairs were analyzed across different cell subpopulations. The relative expression levels (Z-scores) of ligand-receptor pairs and adjusted P values were visualized using dot plots or interaction heatmaps.

### Prediction of transcription factor activity using SCENIC

The Python implementation of SCENIC (v0.12.1; https://github.com/aertslab/pySCENIC) was used to analyze gene regulatory networks (GRNs) and transcription factor (TF) activity under default parameters (Aibar et al., 2017). The normalized expression matrix of the target cells was used as input. During analysis, the RcisTarget and GRNboost transcription factor-binding motif databases for *Homo sapiens* were applied based on official resources (https://pyscenic.readthedocs.io/en/latest). Regulon-specificity scores were calculated through the SCENIC workflow, and active transcription factors were identified from the binarized matrix. In addition, based on the AUC matrix, differentially active transcription factors were screened using the Wilcoxon test, with thresholds of FDR <0.05 and fold change >2. The final results were visualized as heatmaps.

### CytoTRACE analysis

The CytoTRACE algorithm, developed by Gulati et al., is an advanced computational tool for scRNA-seq analysis that captures, refines, and quantifies gene expression features strongly correlated with the number of genes expressed per cell. After CytoTRACE computation, each cell is assigned a score reflecting its stemness or differentiation status within a given dataset. As a robust computational approach, CytoTRACE has been shown to accurately infer cellular differentiation states from scRNA-seq data and has demonstrated superior performance over conventional stemness assessment algorithms in large-scale datasets (Kang et al., 2025). In this study, CytoTRACE scores of the target cells were calculated using the CytoTRACE R package (v0.3.3), with values ranging from 0 to 1. Higher scores indicate lower differentiation states, whereas lower scores indicate more differentiated cellular states.

### Pseudotime trajectory analysis

Monocle2 (v2.20.0) was used for pseudotime trajectory analysis to infer developmental differentiation trajectories of cells (Qiu et al., 2017). After extracting the UMI count matrix from the Seurat object, a CellDataSet object was created using the newCellDataSet function. Genes with a mean expression level greater than 0.1 were selected for trajectory analysis. Dimensionality reduction was then performed using the DDRTree method, and cells were ordered along the pseudotime trajectory using the orderCells function.

### Differential expression analysis

Differential gene expression analysis was performed using the eBayes function in the limma R package (v3.52.3). Genes with |log2FC| > 1 and FDR <0.05 were considered significantly differentially expressed. Because scRNA-seq data are inherently sparse and affected by dropout events, a relatively moderate log2FC threshold was used to capture cell-cluster and mast-cell-subcluster marker genes with biologically meaningful cell-state specificity. In contrast, for bulk RNA-seq analysis, a stricter |log2FC| > 1 threshold was applied to identify robust sample-level DEGs with larger effect sizes for downstream molecular subtype characterization, PPI network construction, and hub gene screening.

### Functional annotation and gene set enrichment analysis

Functional annotation was conducted based on the Kyoto Encyclopedia of Genes and Genomes (KEGG) database. Significantly enriched functional categories were identified using Fisher’s exact test, and pathways with q values ≤0.05 were considered statistically significant. In addition, gene set enrichment analysis (GSEA) was performed to annotate biological processes and signaling pathways, and normalized enrichment scores (NESs) were calculated. The NES values of statistically significant gene sets were ultimately visualized.

### Construction of the PPI network

The STRING database is an online platform for analyzing known proteins and predicting protein-protein interaction (PPI) networks, including both direct and indirect interactions as well as functional associations among proteins (Szklarczyk et al., 2019). Analysis of molecular interactions and PPI networks facilitates the identification of potential molecular targets, signaling pathways, metabolic pathways, and network functions involved in disease progression. Therefore, in this study, the STRING database was used to construct the PPI network.

### Statistical analysis

The surv_cutpoint function in the survminer R package (v0.4.9) was used to determine the optimal cutoff values for patient stratification. Kaplan-Meier survival curves were generated using the survminer and survival R packages (v3.3–1), and differences between groups were assessed using the two-sided log-rank test. The rms R package (v6.3-0) was used to construct the nomogram model and calibration curves. Decision curve analysis (DCA) was performed using the ggDCA R package (v1.2) to evaluate the clinical benefit of different subtypes. All statistical analyses were conducted using R software (v4.2.0), and a two-sided P value <0.05 was considered statistically significant.

## Results

### Single-cell atlas of the RA microenvironment

**FIGURE 1:**
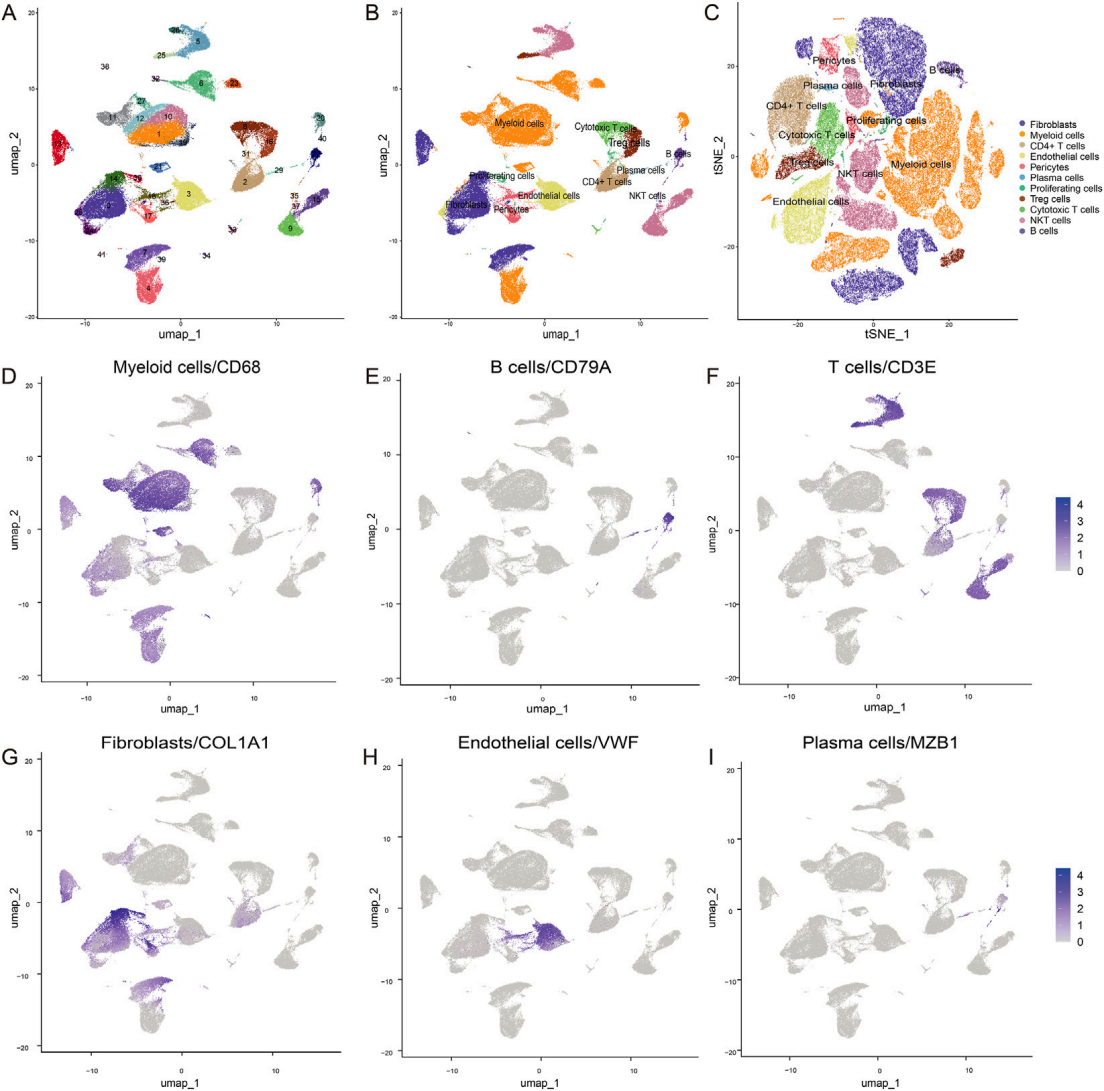
Global landscape of cellular heterogeneity revealed by scRNA-seq in RA. **(A)** UMAP plot showing the dimensionality reduction and clustering of all cells, with 33 distinct cell clusters labeled in different colors. **(B)** UMAP visualization of the major annotated cell types. **(C)** t-SNE plot showing the distribution of annotated cell types. **(D–I)** Differential expression patterns of marker genes for myeloid cells, B cells, T cells, fibroblasts, endothelial cells, and plasma cells, respectively.

We analysed 10x scRNA-seq data, comprising 17 RA samples. To eliminate batch effects among samples, the datasets were integrated to ensure minimal inter-patient differences in the major cell-type features. After quality control and filtering, a total of 104,806 immune cells were retained for unsupervised clustering analysis, which ultimately identified 33 cell clusters (Figure 1A). Based on canonical marker genes, 11 major cell populations were successfully annotated, including CD4^+^ T cells, cytotoxic T cells, regulatory T cells, NKT cells, B cells, plasma cells, myeloid cells, fibroblasts, pericytes, and endothelial cells (Figures 1B,C). In addition, representative marker genes for six major cell types were displayed (Figures 1D–I), including CD68 for myeloid cells, CD79A for B cells, CD3E for T cells, COL1A1 for fibroblasts, VWF for endothelial cells, and MZB1 for plasma cells.

### Subpopulation characteristics of myeloid cells

**FIGURE 2:**
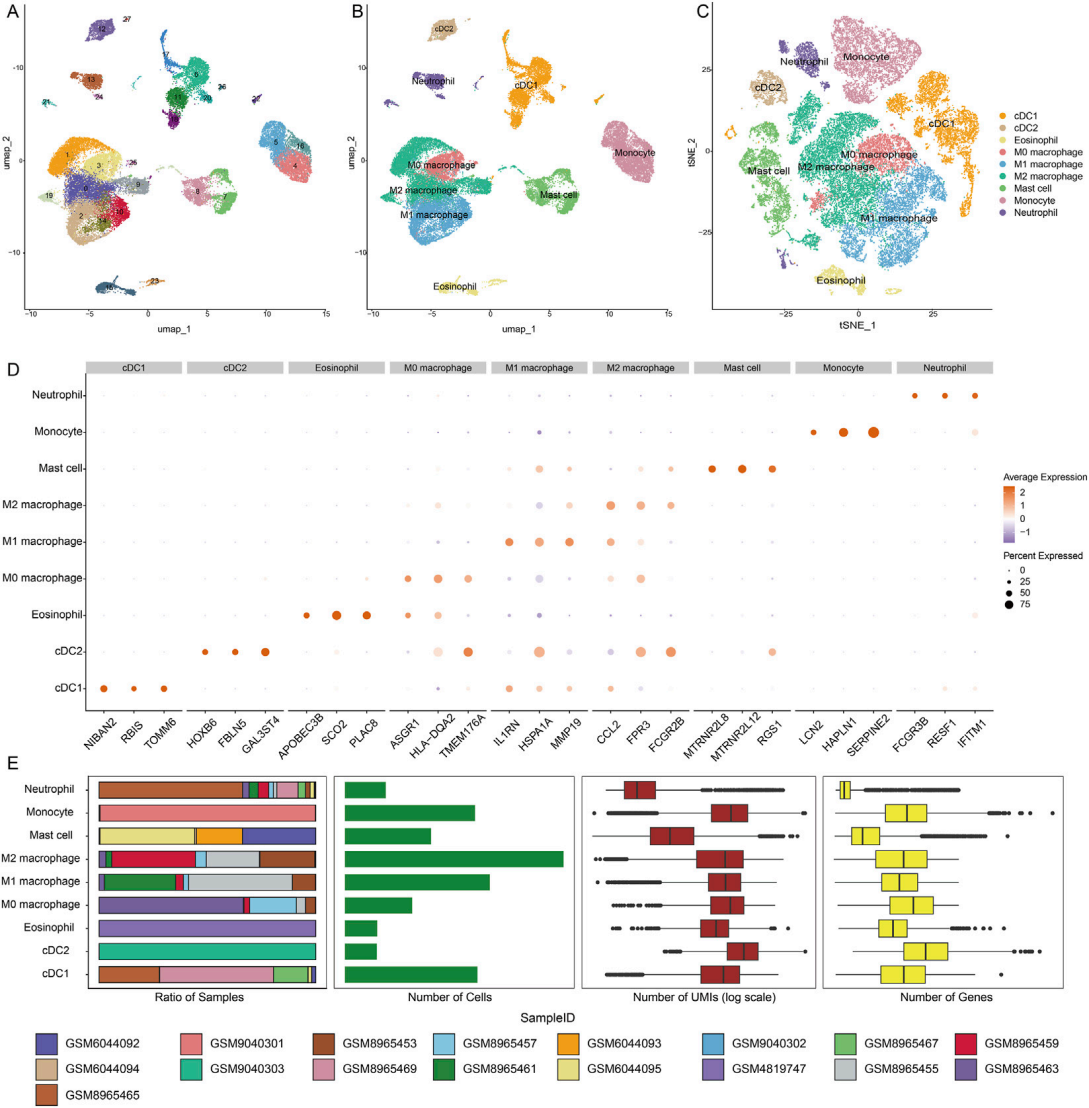
Heterogeneity of myeloid cell subpopulations. **(A)** UMAP plot of myeloid cells showing the distribution of unannotated clusters, with each cluster labeled by a distinct color and number. **(B)** UMAP plot of annotated myeloid cell subpopulations, showing the distribution of different subsets, including dendritic cells, monocytes, macrophages, and others. **(C)** Marker gene expression patterns across myeloid cell subpopulations. **(D)** Bubble plot showing differential expression of marker genes among myeloid cell subsets. **(E)** Overview of the basic features of myeloid cell subpopulations. From left to right: proportional distribution of each subset across samples, cell number statistics for each subset, distribution of UMI counts, and distribution of detected gene numbers in each subset.

Through unsupervised clustering analysis, 36,114 myeloid cells were divided into 28 clusters (Figure 2A), which were further classified into nine major cell subtypes (Figures 2B,C), including monocytes, M0 macrophages, M1 macrophages, M2 macrophages, conventional dendritic cell 1 (cDC1), conventional dendritic cell 2 (cDC2), neutrophils, eosinophils, and MCs. Each cell subtype exhibited distinct molecular characteristics (Figure 2D). Among them, MCs formed a clearly separated and relatively independent cluster and specifically expressed marker genes such as MTRNR2L8, MTRNR2L12, and RGS1, suggesting a unique transcriptomic profile. Further analysis showed that although MCs did not account for a dominant proportion overall, they exhibited relatively high UMI counts and gene numbers (Figure 2E), indicating abundant transcriptomic features and well-defined cellular states. These findings suggest that MCs can not only be stably identified in the myeloid-cell atlas, but also possess distinct molecular characteristics and substantial biological relevance. To further support the identity of the annotated MC population, we examined the expression of canonical mast cell markers, including TPSAB1, CPA3, KIT, MS4A2, and TPSB2. These markers were consistently enriched in the MC cluster, whereas markers associated with monocytes, macrophages, dendritic cells, and other myeloid populations showed distinct expression patterns. This marker profile supports the reliable identification of MCs within the RA synovial myeloid-cell atlas.

### Heterogeneity of mast cells in RA

**FIGURE 3:**
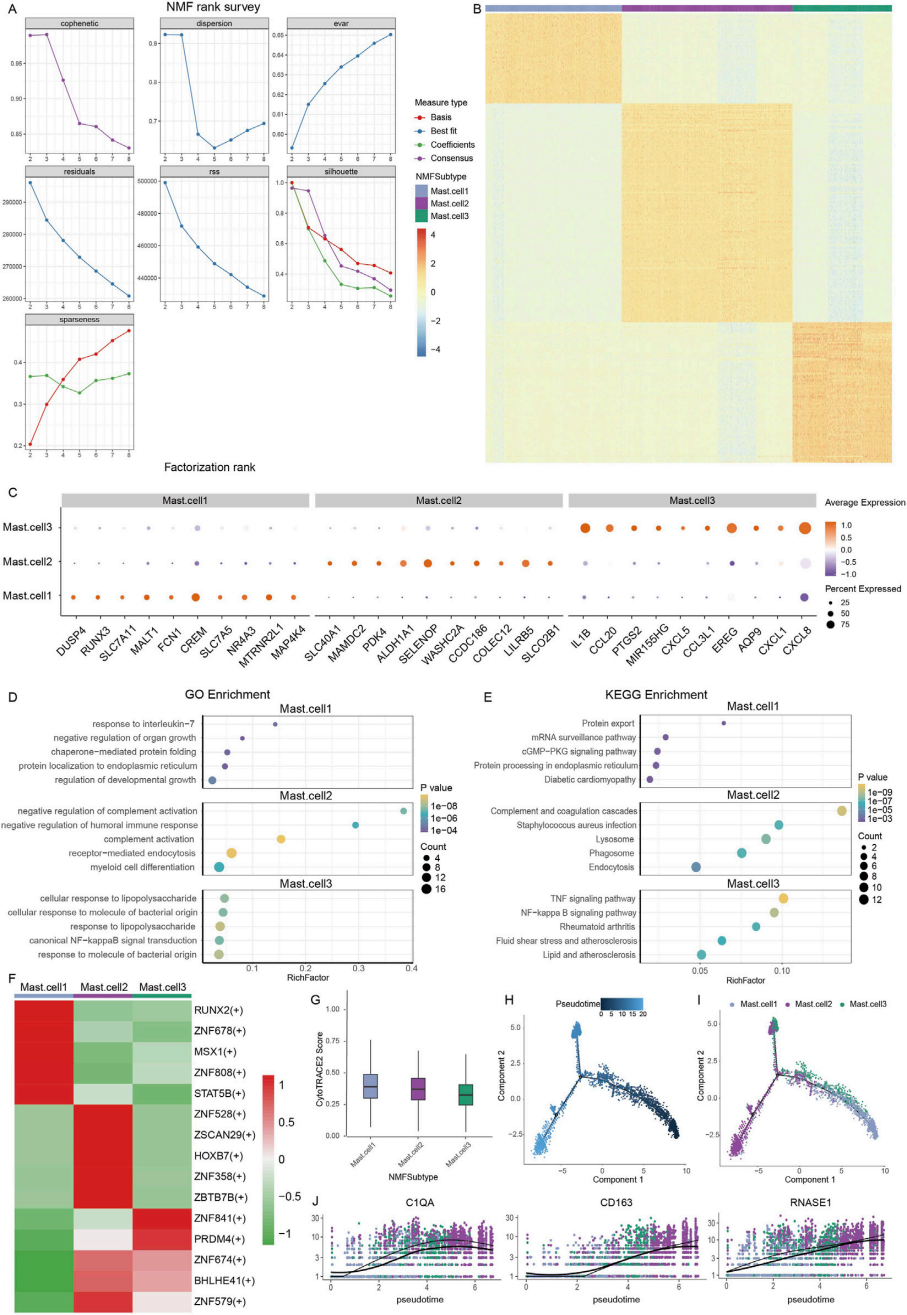
Clustering analysis of mast cell subpopulations. **(A)** Relationship between clustering coefficient metrics and the number of MC clusters (k) in NMF analysis. **(B)** Heatmap showing the differentially expressed genes among the 3 MC subpopulations. **(C)** Bubble plot showing differential expression of marker genes among the three MC subpopulations. **(D)** Bubble plot showing significantly enriched GO terms for each MC subpopulation. **(E)** Bubble plot showing significantly enriched KEGG pathways for each MC subpopulation. **(F)** Heatmap of subtype-specific transcriptional regulatory networks identified by SCENIC analysis, showing the activities of key transcription factors across the 3 MC subpopulations. **(G)** CytoTRACE2 analysis showing differences in differentiation potential among the 3 MC subpopulations. **(H)** Monocle2 pseudotime trajectory analysis showing the transitional trajectories of different MC subpopulations; the color gradient represents pseudotime progression. **(I)** Annotation of MC subpopulations along the pseudotime trajectory. **(J)** Dynamic changes in the expression of marker genes from the three MC subpopulations along pseudotime.

Given the distinct molecular characteristics of MCs, we next explored their functional diversity from the perspective of intrapopulation heterogeneity. First, non-negative matrix factorization was applied to cluster 3,515 MCs, and k = 3 was identified as the optimal number of clusters (Figure 3A). Accordingly, MCs were divided into three subpopulations: Mast cell 1 (1,179 cells), Mast cell 2 (1,476 cells), and Mast cell 3 (860 cells). All three MC subpopulations retained the expression of core mast cell-associated markers, supporting their shared MC identity. Meanwhile, each subset exhibited distinct subtype-specific transcriptional features, indicating that these clusters likely represent different functional states within the MC compartment rather than separate non-MC populations or annotation artifacts. To identify subtype-specific marker genes, differential expression analysis was performed, and marker genes were screened using the thresholds avg_log2FC > 0.5, pct.1 > 0.4, and pct.2 < 0.6. The results showed that Mast cell 1 had 62 specific marker genes, Mast cell 2 had 162, and Mast cell 3 had 91 (Figure 3B). We further displayed the top 10 differentially expressed genes in each subgroup (Figure 3C), including CREM in Mast cell 1, SELENOP in Mast cell 2, and IL1B in Mast cell 3.

We then performed GO and KEGG enrichment analyses for the subtype-specific marker genes of each mast cell subpopulation, which revealed pronounced functional heterogeneity among these subsets. Mast cell 1 was mainly enriched in functions related to protein modification and growth regulation, such as negative regulation of organ growth and protein lipoylation-mediated protein folding (Figure 3D); at the pathway level, it was enriched in protein-processing and RNA metabolism-related pathways, including Protein processing in endoplasmic reticulum and mRNA surveillance pathway (Figure 3E). Mast cell 2 was primarily enriched in immune- and inflammation-regulatory functions, such as negative regulation of complement activation and regulation of inflammatory response (Figure 3D); the corresponding enriched pathways included Complement and coagulation cascades and *Staphylococcus aureus* infection (Figure 3E). Mast cell 3 was mainly enriched in pathogen-response-related functions, such as cellular response to lipopolysaccharide and response to molecule of bacterial origin (Figure 3D); at the pathway level, it was enriched in inflammation- and signal transduction-related pathways, including the TNF signaling pathway and NF-kappa B signaling pathway (Figure 3E).

Further SCENIC analysis revealed highly subtype-specific transcription factor activities across the three MC subsets (Figure 3F). In Mast cell 1, transcription factors such as RUNX2 and ZNF678 showed high activity, suggesting potential roles in promoting cell proliferation. In Mast cell 2, STAT5B and ZNF528 were markedly activated and may serve as subtype-specific regulators. In Mast cell 3, transcription factors such as ZNF841 and PRDM4 exhibited high activity and may underlie the characteristic functional properties of this subset. In addition, CytoTRACE analysis was used to assess the differentiation potential of the three mast cell subpopulations. The results showed a progressively decreasing trend in CytoTRACE scores across the three subsets, suggesting that Mast cell 1 may differentiate into Mast cell 2 and Mast cell 3 (Figure 3G). Pseudotime trajectory analysis further supported the developmental relationship among these three subpopulations (Figures 3H,I). Meanwhile, the expression levels of subtype-specific marker genes changed dynamically along pseudotime (Figure 3J).

**FIGURE 4:**
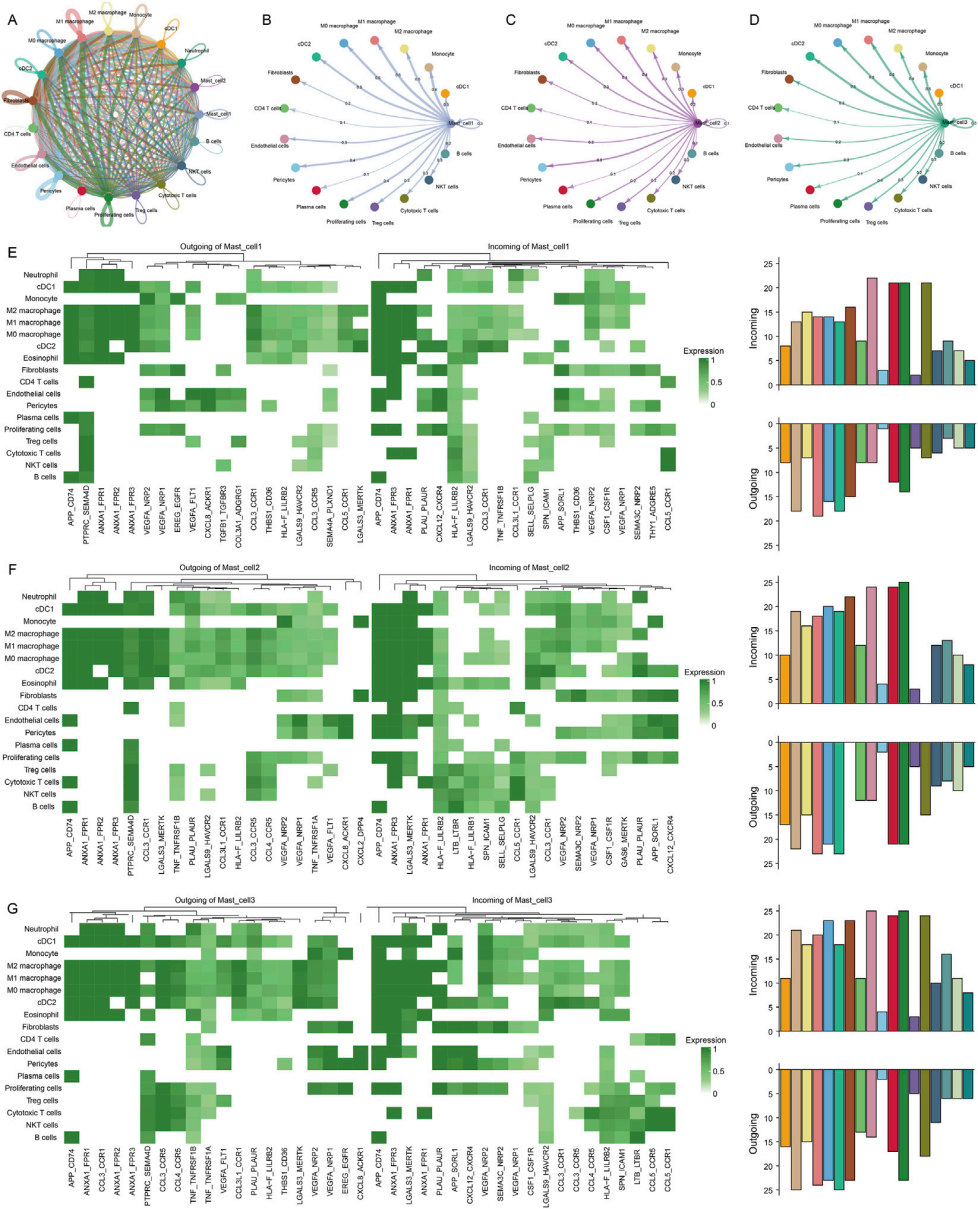
Cell-cell interaction analysis of mast cell subpopulations. **(A)** Global interaction network of all cell populations in the microenvironment. Nodes represent different cell types (such as M1 macrophages, fibroblasts, and MC subpopulations), and edges represent ligand-receptor interactions between cells. **(B–D)** Subtype-specific interaction networks for each MC subpopulation. **(E–G)** Ligand-receptor interaction heatmaps and interaction strength statistics for each MC subpopulation.

Cell-cell communication analysis showed that all three MC subtypes displayed extensive interactions with multiple cell populations, particularly with M0/M1/M2 macrophages, monocytes, and cDC2 cells (Figures 4A–D), suggesting close associations between mast cells and myeloid immune cells. Further ligand-receptor interaction heatmap analysis demonstrated that all three MC subtypes exhibited abundant incoming and outgoing signals (Figures 4E–G), indicating that they are capable of both sensing microenvironmental cues and actively modulating the states of surrounding cells. Compared with Mast cell 1, Mast cell 2 and Mast cell 3 displayed a broader range and greater number of interactions overall, with Mast cell 3 showing the most extensive interaction spectrum, suggesting that it may represent a more functionally active MC subset. These interactions mainly involved inflammatory regulation, chemotactic recruitment, cell adhesion, and vascular/stromal remodeling, indicating that distinct MC subtypes may participate in microenvironmental regulation through differential communication patterns.

### Identification of mast cell-related molecular subtypes

**FIGURE 5:**
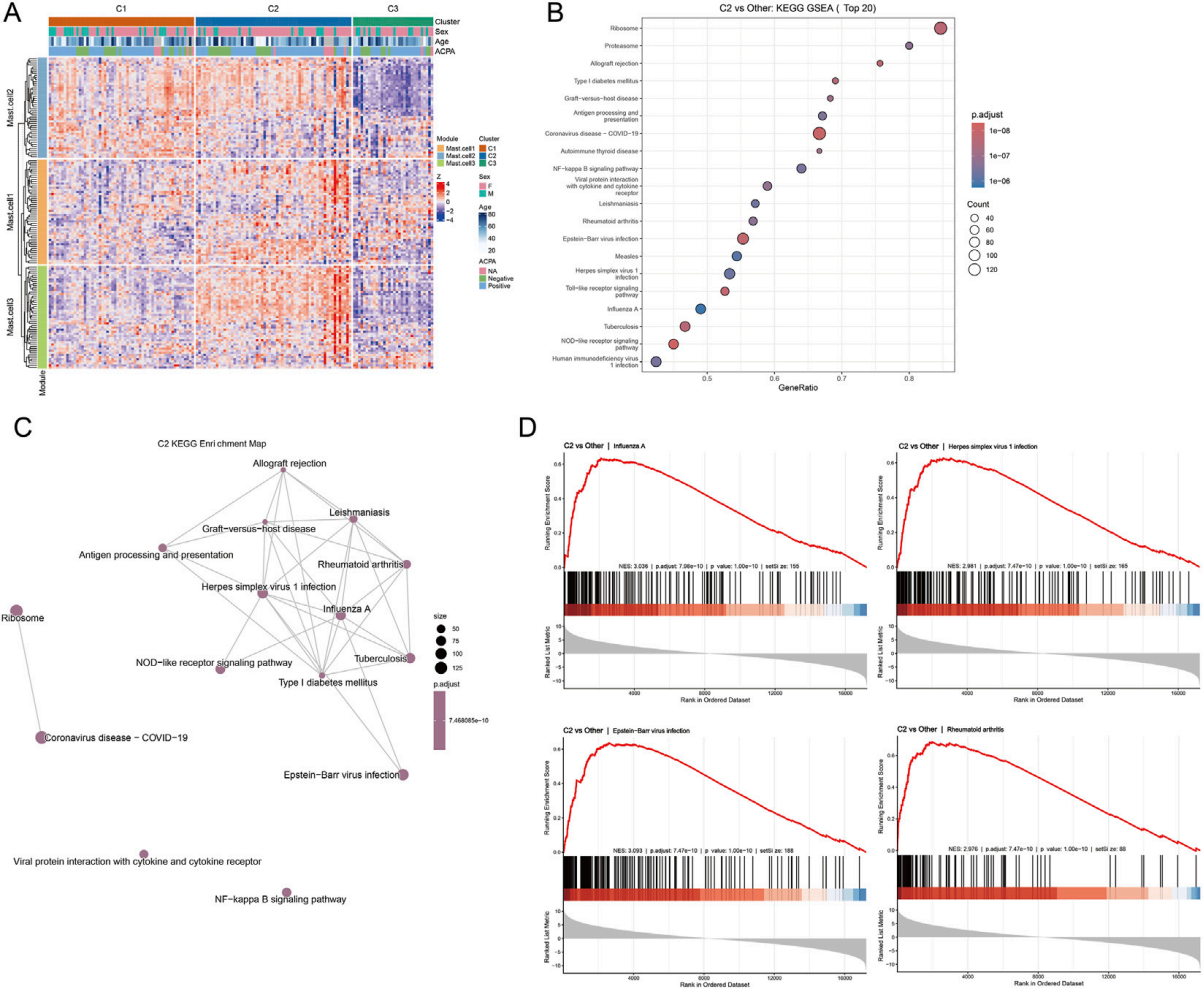
Identification of mast cell-related molecular subtypes in RA and enrichment landscape across subtypes. **(A)** Consensus clustering heatmap showing unsupervised classification of 218 RA synovial samples based on MC signature gene sets. **(B)** Bubble plot summarizing the top significantly enriched KEGG pathways in subtype C2. **(C)** Enrichment network showing the functional organization and interconnections among pathways enriched in subtype C2. **(D)** GSEA plots showing detailed enrichment curves for four representative pathways, including Epstein-Barr virus infection, Rheumatoid arthritis, NF-kappa B signaling pathway, and Chemokine signaling pathway.

To systematically characterize the molecular heterogeneity of RA, we included transcriptomic data from synovial tissues of 218 patients with RA in the HMU cohort. Unsupervised consensus clustering based on the three MC signature gene sets stratified the cohort into three stable molecular subtypes, that is C1-C3 (Figure 5A). The annotation bar above the heatmap showed that C2 exhibited a distinct high-infiltration phenotype, with significantly elevated Mast. cell1 and Mast. cell3 signature scores compared with the other two groups, suggesting that this subtype is strongly associated with MC activity.

GSEA further defined the biological states of each subtype. KEGG pathway enrichment analysis revealed widespread activation of RA-specific pathogenic mechanisms in the C2 subtype (Figure 5B). Notably, this highly inflammatory feature was strongly subtype-specific. In contrast to the extensive activation observed in C2 subtype, both C1 and C3 subtypes showed significant negative enrichment for inflammatory and virus-related pathways under the same analytical framework, defining them as relatively low-risk, immune-quiescent subtypes (Supplementary Figures 1A–F).

Among the most highly enriched pathways in the C2 subtype, Epstein-Barr virus infection and Rheumatoid arthritis were dominant, suggesting a potential link between viral infection-related programs and autoimmune pathology. To clarify the intrinsic relationship among these pathways, we constructed a pathway interaction network (Figure 5C). The topological structure of this network showed that viral infection-related pathways (such as EBV and herpes simplex) were tightly intertwined with inflammatory pathways (such as NF-kappa B signaling) and RA autoimmune pathways, forming a highly cohesive functional module.

Further detailed GSEA analyses of core pathways confirmed the robustness of these mechanisms, Epstein-Barr virus infection, Rheumatoid arthritis, and Chemokine signaling pathway all exhibited significant unimodal enrichment patterns in the C2 subtype (Figure 5D). Collectively, these findings indicate that C2 subtype represents a high-risk subtype with strong inflammatory activity and canonical RA pathological features, potentially driven in part by viral mimicry-related signaling.

### Distinct immune infiltration landscape of the high-risk subtype

**FIGURE 6:**
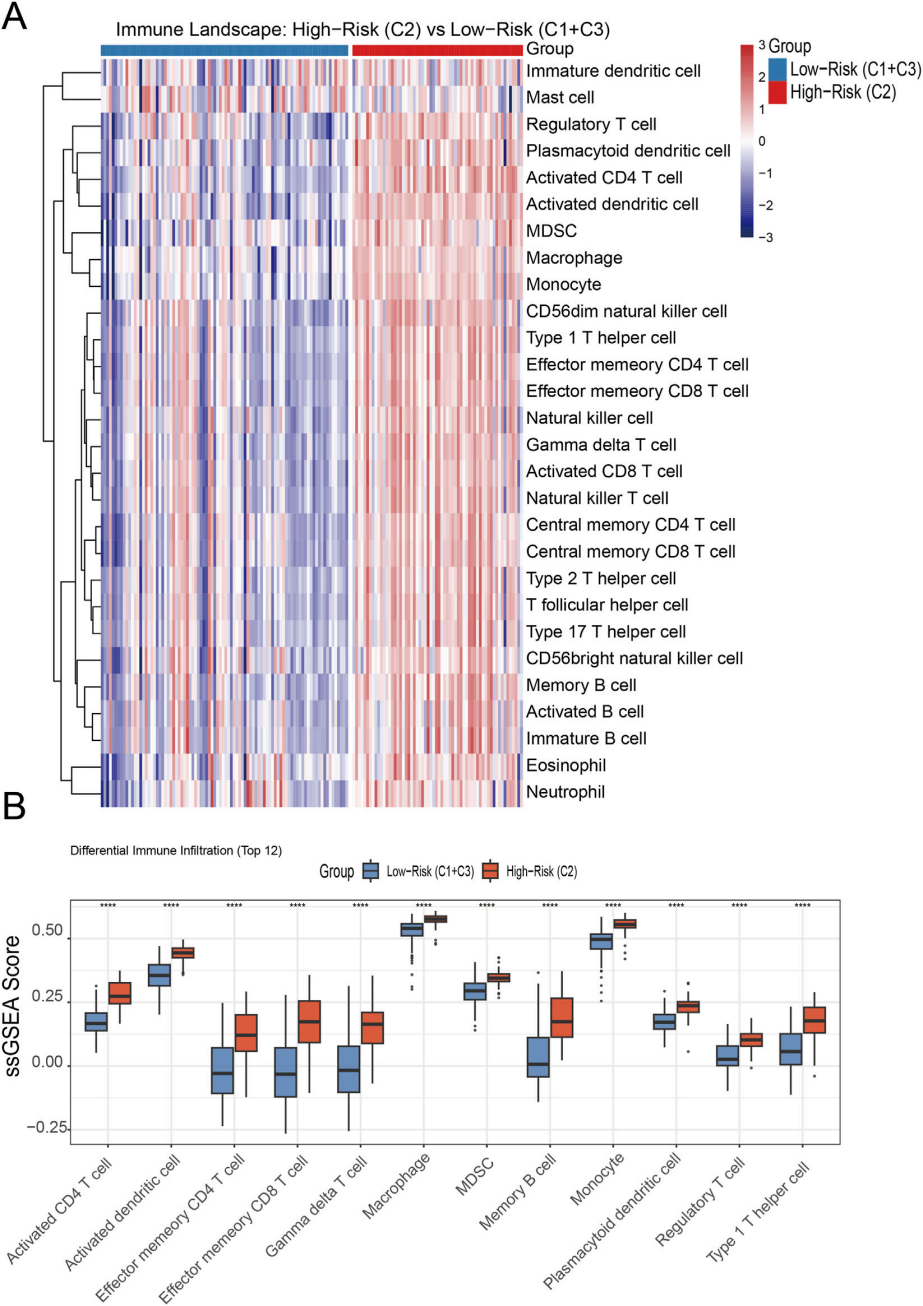
Distinct immune infiltration pattern of the high-risk subtype. **(A)** Heatmap of immune infiltration based on unsupervised clustering of 28 immune cell types quantified by ssGSEA in the HMU cohort. **(B)** Boxplots showing differentially infiltrated immune cell populations between the high-risk and low-risk groups.

To systematically characterize microenvironmental heterogeneity, we divided patients into a High-Risk group (C2) and a Low-Risk group (C1 + C3), and used ssGSEA to quantify the relative abundance of 28 immune cell populations. The immune landscape heatmap clearly revealed markedly distinct microenvironmental patterns between these two groups (Figure 6A). The High-Risk group exhibited a typical immune-hot phenotype, characterized by extensive immune-cell infiltration, whereas the Low-Risk group displayed an immune-cold state. Differential statistical analysis further confirmed that most pro-inflammatory and effector immune cell populations were significantly elevated in the High-Risk group (Figure 6B). Specifically, adaptive immune cells such as activated CD4 T cells and memory B cells, as well as innate immune cells such as macrophages, monocytes, and activated dendritic cells, were markedly enriched. The simultaneous increase in myeloid-derived suppressor cells (MDSCs) suggested the presence of a complex immunoregulatory feedback mechanism within this microenvironment.

Of note, although this subtype was originally defined based on MC signature gene sets, ssGSEA analysis showed that the relative abundance of MCs themselves did not differ significantly between groups (High-Risk: 0.12 vs. Low-Risk: 0.15, P = 0.221; Supplementary Table S1). This finding suggests that the high-risk nature of the C2 subtype is not simply driven by an increase in MC number, but may instead result from the recruitment of large numbers of T cells and macrophages by chemokines secreted from highly activated MCs (such as CXCL13 and CCL2), thereby generating an immune-masking effect in which MCs no longer dominate proportionally within the overall infiltrating immune landscape.

### Identification of subtype-specific signatures and the proliferation-inflammation driver network

**FIGURE 7:**
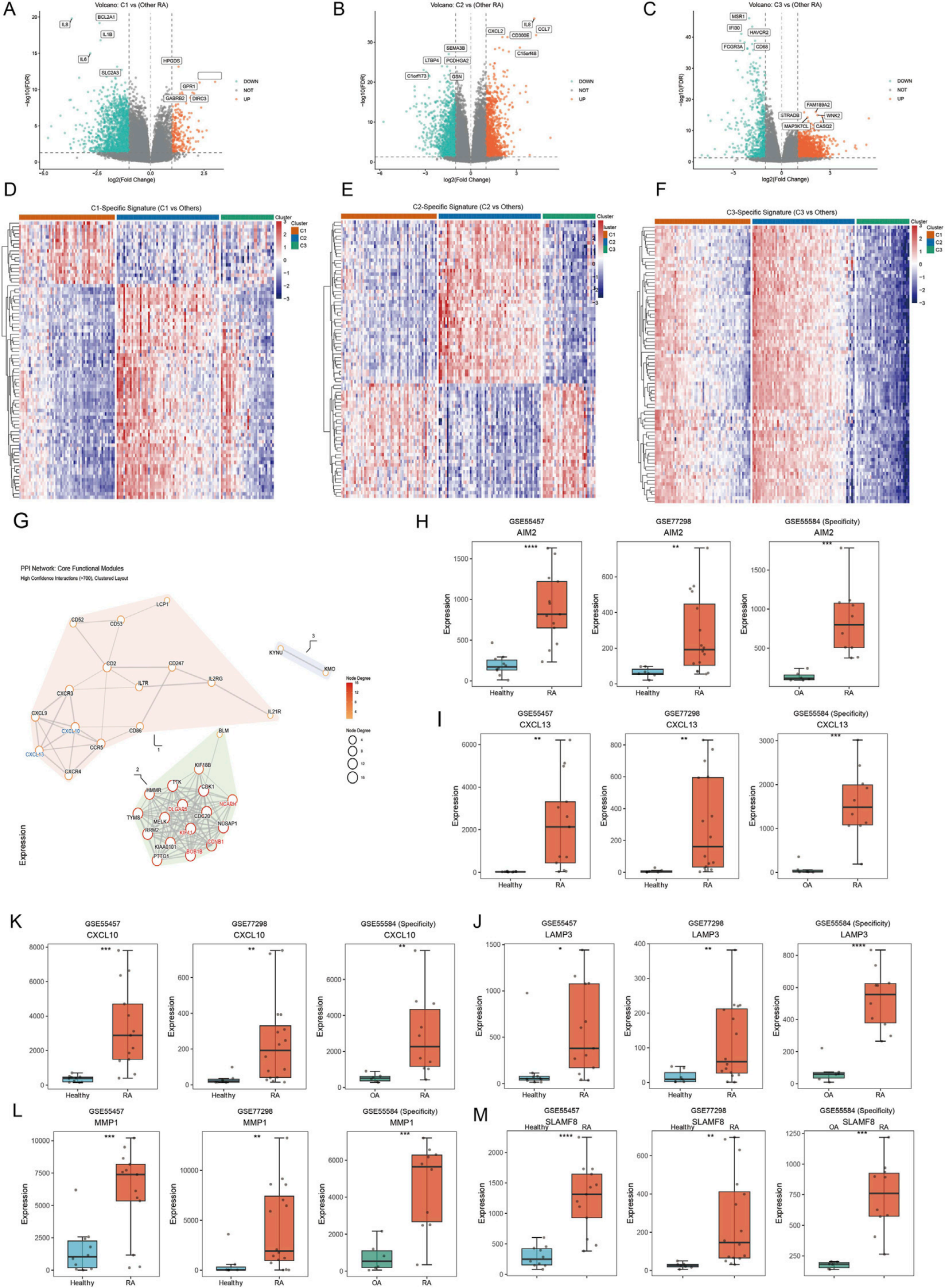
Landscape of subtype-specific signatures and validation of the proliferation-inflammation driver network. **(A–C)** Volcano plots showing subtype-specific upregulated genes identified by pairwise comparisons among C1, C2, and C3 relative to the other subtypes. **(D–F)** Heatmaps showing subtype-specific gene expression patterns. **(G)** PPI network of robust core genes specific to subtype C2. **(H–M)** Validation of hub genes. Boxplots from the independent validation cohort (GSE55235) confirmed their clinical relevance. The identified hub genes (CCNB1, NCAPH, and DLGAP5) were significantly upregulated in patients with RA compared with healthy controls (P < 0.05), supporting their roles as strong pathogenic drivers beyond nonspecific inflammatory responses.

To comprehensively define the molecular features of the C2 high-risk subtype, we first performed multi-group differential expression analysis in the discovery cohort. The volcano plots showed that the C2 subtype exhibited the most dramatic transcriptomic reprogramming (Figures 7A–C). Subtype-specific heatmaps further confirmed the explosive upregulation of key pathogenic mediators in C2, including MMP1, MMP10, CXCL13, and IL8 (Figures 7D–F). These findings established the molecular identity of C2 as a high-inflammatory subtype.

To identify the core mechanisms driving this phenotype, we constructed a PPI network consisting of 173 robust genes (Figure 7G). Topological analysis revealed a highly cohesive functional module, the center of which was dominated by cell-cycle regulators. The top three hub genes (red nodes), CCNB1, NCAPH, and DLGAP5, formed a tightly connected regulatory core. In contrast, inflammatory effectors (blue nodes), such as the highly expressed chemokine CXCL13, were distributed mainly at the periphery of the network. This topological architecture suggested a dual-driver mechanism underlying the C2 high-risk subtype: on one hand, broad immune-cell recruitment mediated by chemokines such as CXCL13; on the other hand, persistent synovial fibroblast proliferation driven by key cell-cycle regulators such as CCNB1 and NCAPH. Together, these processes may synergistically promote severe pannus formation and joint destruction.

To validate the clinical relevance of these hub genes, we evaluated their expression patterns across independent cohorts and subtypes (Figures 7H–M). First, in the GSE55235 validation cohort (healthy vs. RA), DLGAP5 (mean: 33.32 vs. 12.43, P < 0.001) and CCNB1 (P = 0.043) were significantly upregulated, supporting their potential as general pathogenic factors in RA. Interestingly, NCAPH did not show a statistically significant difference in this overall comparison (P > 0.05).

To further confirm the robustness and specificity of these biomarkers, we extended the validation to a broader set of datasets, including GSE55457, GSE77298, and the osteoarthritis control cohort GSE55584. After rigorously excluding non-specific interference through RA-versus-OA comparisons, CCNB1, NCAPH, and DLGAP5 were all found to be significantly and specifically upregulated in RA samples. This multi-cohort, cross-platform validation strongly supports that these cell-cycle-related hub genes are key drivers of RA-specific synovial hyperplasia. In particular, NCAPH may serve as a critical molecular discriminator between high-risk and low-risk RA subtypes. Targeting these upstream cell-cycle drivers may therefore offer a more fundamental therapeutic strategy than merely blocking downstream inflammatory mediators.

### Construction and evaluation of a subtype-specific clinical prediction model

**FIGURE 8:**
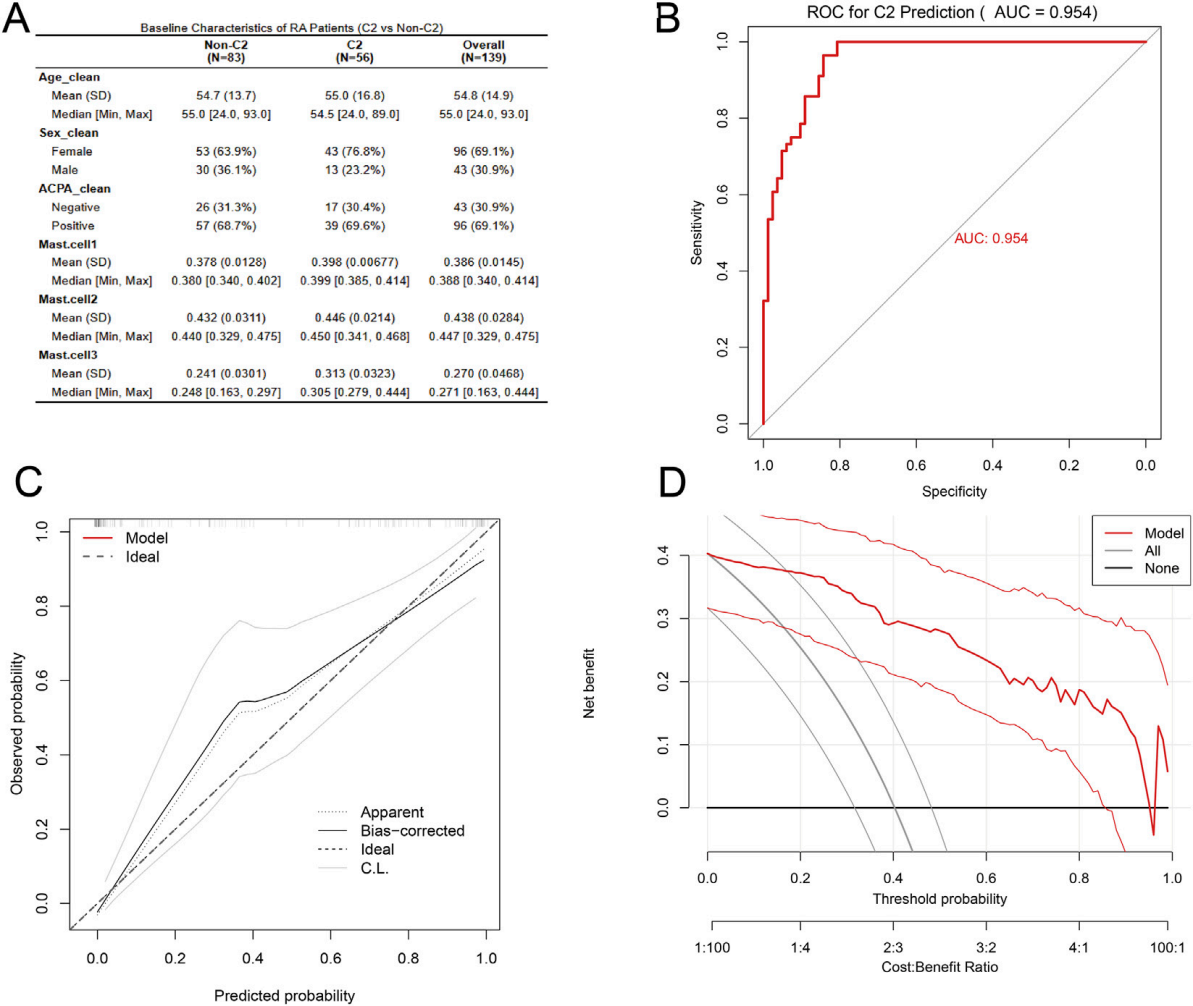
Construction and validation of a diagnostic nomogram for the high-risk C2 subtype. **(A)** Diagnostic nomogram. **(B)** Receiver operating characteristic curve. **(C)** Calibration curve. **(D)** Decision curve analysis.

Given the high-risk pathological features of the C2 subtype, the development of a practical clinical tool for identifying patients with this subtype is of considerable importance. Using multivariable logistic regression analysis, we constructed a diagnostic nomogram based on MC scores (Figure 8A). Regression analysis showed that Mast. cell1 and Mast. cell2 were independent and significant predictive factors, whereas conventional clinical characteristics, such as age and ACPA status, did not reach statistical significance in the model. These results suggest that MC scores derived from transcriptomic features may be more sensitive than traditional clinical indicators for subtype identification.

Performance evaluation demonstrated the excellent accuracy and utility of this predictive tool. ROC analysis showed that the AUC reached 0.954, indicating excellent discriminative ability for identifying high-risk C2 patients from the broader RA population (Figure 8B). The calibration curve demonstrated high consistency between predicted and observed probabilities (Figure 8C). Bootstrap validation (B = 1,000) yielded a mean absolute error of only 0.045, suggesting that the model provided highly accurate risk estimation without obvious overfitting or underestimation. Decision curve analysis (Figure 8D) further confirmed the clinical utility of the model. Across a broad threshold probability range of 1%–90%, the net benefit of using this nomogram for decision-making (red line) consistently exceeded that of either the “treat-all” or “treat-none” strategies. These findings indicate that risk stratification based on this model may maximize clinical benefit without increasing the cost of misclassification.

## Discussion

In the present study, by integrating single-cell and bulk transcriptomic data, we systematically characterized the heterogeneity of MCs within the RA synovial immune microenvironment and further established a MC-related molecular classification and risk prediction model. Our findings indicate that MCs in RA synovium are not homogeneous inflammatory participants, but rather consist of multiple subpopulations with distinct transcriptional programs, functional states, and cell-cell communication capacities. These subpopulations not only exhibited marked differences at the single-cell level, but also delineated RA molecular subtypes with distinct inflammatory intensity and immune infiltration patterns at the bulk tissue level. Collectively, these findings suggest that the role of MCs in RA may be driven more by functional state than by simple quantitative changes, thereby providing a new perspective for understanding the complexity of the RA synovial immune microenvironment. More broadly, RA pathogenesis involves not only immune-cell infiltration and cytokine-mediated inflammation, but also multiple interconnected mechanisms that promote loss of tolerance and persistent synovial immune activation. Autophagy has been proposed to participate in the generation of post-translationally modified autoantigens, including citrullinated and carbamylated peptides, thereby contributing to autoreactive immune responses. In addition, secretory autophagy and extracellular vesicles may mediate intercellular immune communication by transferring post-translationally modified proteins, inflammatory mediators, and RNA species within the synovial microenvironment. In this broader context, the MC-related inflammatory and communication programs identified in our study may represent one component of a larger pathogenic network linking innate immune activation, autoantigen generation, extracellular vesicle-mediated signaling, stromal remodeling, and persistent autoimmune inflammation in RA. (Riitano et al., 2023).

Previous studies on RA pathogenesis have mainly focused on classical effector cells such as T cells, B cells, macrophages, fibroblast-like synoviocytes, and dendritic cells, whereas although MCs have long been considered contributors to inflammatory mediator release and synovial damage, their fine-grained stratification and precise functional roles in RA remain poorly defined (McHugh, 2024). Recent experimental work by Lei et al. further demonstrated that the RA synovial microenvironment can shape MC activation, including MRGPRX2-associated degranulation and upregulation of MHC class II and costimulatory molecules, thereby supporting a pathogenic role of MCs in regulating T-cell responses and releasing inflammatory mediators in RA (Lei et al., 2024). Our findings are broadly consistent with this concept, as all three MC subsets showed distinct immune-regulatory features and extensive communication with other immune-cell populations. However, the present study extends previous work by resolving RA synovial MCs into three transcriptionally distinct functional states and linking these MC subset signatures to bulk transcriptomic molecular subtypes. In this framework, Mast cell 3, characterized by TNF/NF-κB signaling activation, may partially correspond to an inflammation-induced activated MC state similar to that described by Lei et al., whereas Mast cell 1 and Mast cell 2 may represent relatively upstream/metabolic and complement/inflammation-regulatory states, respectively. Therefore, our proposed MC classification should be viewed as a complementary transcriptomic stratification rather than a direct replacement of previously described MC activation phenotypes. In this study, we first identified a stably existing MC population in the integrated single-cell dataset from 17 RA samples and found that MCs formed a relatively independent transcriptional cluster within the myeloid-cell atlas. Moreover, this population exhibited relatively high UMI counts and gene counts, suggesting that MCs are not merely low-abundance bystander cells, but may instead possess high transcriptional activity and clearly defined biological states. These findings support the view that MCs are an important component of the RA synovial immune microenvironment and further underscore the necessity of in-depth characterization using single-cell approaches (Rivellese et al., 2019; Rivellese et al., 2018).

Further analysis using non-negative matrix factorization divided MCs into three subpopulations with distinct functional tendencies. The biological relevance of these MC subsets was supported by multiple complementary lines of evidence. In addition to the retained expression of canonical MC markers, the three subsets showed distinct marker-gene profiles, pathway-enrichment patterns, transcription factor activities, inferred differentiation/activation trajectories, and cell-cell communication networks. These findings suggest that the identified MC subsets reflect biologically meaningful functional states within the RA synovial microenvironment rather than technical clustering artifacts. Moreover, the use of MC subset signature genes for molecular stratification in the bulk RNA-seq cohort further linked these single-cell-defined states to tissue-level inflammatory phenotypes. Mast cell 1 was primarily associated with protein processing, RNA surveillance, and growth regulation; Mast cell 2 was closely related to complement regulation and inflammatory response modulation; and Mast cell 3 was significantly enriched in lipopolysaccharide response, response to bacterial stimuli, and classical inflammatory pathways such as TNF and NF-kB signaling. These observations suggest that MCs in RA synovium are not in a uniform activation state, but may instead exist along a dynamic continuum ranging from an earlier state toward more differentiated immune-regulatory and inflammatory-effector states. CytoTRACE and pseudotime analyses supported the notion that Mast cell 1 may occupy a relatively upstream position, whereas Mast cell 2 and Mast cell 3 may represent more differentiated terminal states with clearer functional specialization. From a biological perspective, these distinct MC states may arise from both intrinsic developmental transitions and extrinsic inflammatory stimulation within the RA synovial microenvironment. In particular, Mast cell 3, which was characterized by enrichment of TNF and NF-κB signaling pathways, may represent an inflammation-induced effector state directly shaped by synovial pro-inflammatory cues such as TNF, IL-1β, TLR-related stimuli, or other cytokine and danger-signal programs. In this scenario, Mast cell 1 may correspond to a relatively upstream or less activated MC state, whereas sustained exposure to inflammatory mediators could drive transition toward Mast cell 2 or Mast cell 3. However, because these inferences are based on transcriptomic trajectory and enrichment analyses, we cannot determine whether Mast cell 3 arises from a specific precursor population or reflects local activation of pre-existing MCs. Future studies using lineage-tracing approaches, spatial transcriptomics, and functional perturbation experiments will be required to clarify the cellular origin and inducing signals of these MC states. Combined with the distinct transcription factor activity patterns identified by SCENIC, these findings suggest that the pathogenic role of MCs in RA may be driven by finely tuned transcriptional regulatory networks, through which different subpopulations undergo functional transitions from homeostatic maintenance to inflammatory amplification.

Notably, cell-cell communication analysis further reinforced the key regulatory role of MCs within the RA microenvironment. All three MC subpopulations exhibited significant interactions with M0/M1/M2 macrophages, monocytes, and cDC2 cells, among which Mast cell 2 and Mast cell 3 displayed broader incoming and outgoing signaling patterns. These findings suggest that MCs may not participate in RA inflammation in isolation, but instead occupy an important node within the myeloid inflammatory network, influencing the recruitment, polarization, and activation of surrounding innate immune cells through the secretion of chemokines, cytokines, and other mediators. However, CellPhoneDB analysis predicts potential ligand-receptor communication based on transcriptomic expression patterns and does not directly demonstrate physical cell-cell interactions or functional regulatory effects. Thus, the inferred MC interactions with macrophages, monocytes, dendritic cells, fibroblasts, or other cell populations should be interpreted as potential communication axes that require experimental validation. In particular, Mast cell 3 displayed the broadest interaction spectrum, which is consistent with its enrichment in inflammatory pathways such as TNF and NF-kB signaling, indicating that this subset may serve as a major executor of synovial inflammatory amplification. Given the central roles of macrophages and dendritic cells in antigen presentation, inflammatory mediator release, and tissue destruction in RA synovium, the cooperative interplay between MCs and these cell populations may constitute a crucial mechanism for sustaining chronic synovitis and promoting joint damage.

At the tissue level, we further classified RA samples into three stable molecular subtypes based on mc subtype-specific signature genes, among which subtype C2 exhibited the most prominent inflammatory phenotype and high-risk features. KEGG and GSEA analyses showed that C2 was broadly enriched in pathways related to Rheumatoid arthritis, NF-kB signaling, Chemokine signaling, and Epstein-Barr virus infection, suggesting a stronger state of inflammatory activation and immune dysregulation in this subtype. Of particular note, virus infection-related pathways and RA pathogenic pathways formed a highly interconnected functional module, which at the transcriptomic level supports the possibility that viral mimicry or infection-related signaling may contribute to immune amplification in RA (Lei et al., 2024). Accordingly, the enrichment of Epstein-Barr virus infection and other viral infection-related pathways should be interpreted as a transcriptomic signature of antiviral-like immune activation or molecular mimicry-related inflammatory programs, rather than direct evidence of active viral infection or pathogen-driven subtype divergence. In this context, the enrichment of Epstein-Barr virus infection and other viral infection-related pathways may reflect a broader “antiviral-like” innate immune hyperactivity rather than direct evidence of active viral infection. Such a transcriptional state may involve coordinated activation of interferon-associated programs, NF-κB signaling, and chemokine-mediated immune recruitment, thereby amplifying autoimmune inflammation in the RA synovium. In addition, molecular mimicry, a recognized concept in autoimmunity, may provide a possible interpretive framework linking antiviral-like immune activation with autoreactive inflammatory responses. Nevertheless, this hypothesis remains speculative and should be validated in future studies through direct pathogen detection, antiviral immune profiling, and functional assays.

One particularly insightful finding was that although the C2 subtype was defined by MC signature genes, ssGSEA analysis showed no significant difference in the relative abundance of MCs themselves between the high- and low-risk groups. In contrast, multiple pro-inflammatory and effector immune cell populations, including activated CD4 T cells, memory B cells, monocytes, macrophages, and activated dendritic cells, were significantly increased in the high-risk group. This suggests that the role of MCs in RA may be reflected primarily in qualitative changes rather than quantitative accumulation; in other words, MCs may shape a more inflammatory immune niche through their activation status and secretory programs, thereby recruiting and amplifying the infiltration and activation of other immune cells (Lei et al., 2024). This interpretation is highly consistent with the extensive myeloid interaction patterns observed in our single-cell communication analysis and may also help explain why previous studies have yielded inconsistent conclusions when evaluating the pathogenic significance of MCs solely on the basis of infiltration abundance. Put differently, the pathogenic relevance of MCs in RA may depend more on specific subpopulations and their functional states than on total MC abundance.

In the high-risk C2 subtype, we further identified a proliferation-inflammation dual-driver network dominated by hub molecules such as CCNB1, NCAPH, and DLGAP5. The PPI network indicated that these core genes were mainly involved in cell-cycle regulation, whereas highly expressed peripheral molecules such as CXCL13, IL8, MMP1, and MMP10 were more representative of inflammatory chemotaxis and matrix-destructive effectors. This network architecture suggests that the development of the RA high-risk subtype is not driven solely by immune-inflammatory mediators, but may instead result from the synergistic effects of aberrant synovial-cell proliferation and inflammatory chemotactic signaling. Specifically, cell-cycle-related hub molecules may provide the proliferative “engine” for pannus formation and persistent synovial hyperplasia, while chemokines and matrix-degrading molecules further promote immune-cell accumulation and cartilage/bone destruction. This model integrates the two central pathological features of RA—inflammation and tissue hyperplasia/invasiveness—and further suggests that MC-related molecular stratification may bridge immune regulation and synovial remodeling (Humby et al., 2019). Mechanistically, MC activity may provide a potential upstream link between inflammatory immune regulation and stromal-cell proliferative remodeling. Our cell-cell communication analysis suggested that MC subsets are extensively connected with immune and stromal-related cell populations, supporting the possibility that activated MCs may influence synovial fibroblasts or other stromal cells through paracrine inflammatory, chemotactic, and tissue-remodeling signals. These MC-derived signals may not directly encode the cell-cycle machinery, but they could create a cytokine-rich and chemokine-rich microenvironment that favors the induction of proliferative programs in recipient stromal cells, thereby contributing to the upregulation of cell-cycle regulators such as CCNB1, NCAPH, and DLGAP5 in the high-risk subtype. However, the current ligand-receptor analysis does not establish a direct regulatory relationship between specific MC-derived ligand-receptor pairs and these intracellular hub genes. Therefore, this proposed MC-stromal proliferation axis should be interpreted as a hypothesis-generating model that requires further validation through MC-fibroblast co-culture experiments, conditioned-medium stimulation, ligand/receptor blockade assays, and spatial transcriptomic analyses. Multi-cohort validation additionally confirmed the upregulated expression trends of CCNB1, NCAPH, and DLGAP5 in RA, supporting that these molecules are not merely statistically associated signals, but may represent biologically meaningful pathogenic drivers.

From a translational perspective, the MC score-based nomogram established in this study demonstrated high discriminative ability and good calibration performance, with an area under the ROC curve of 0.954, indicating its potential utility for identifying the high-risk C2 subtype. Traditional RA risk assessment relies largely on clinical symptoms, autoantibodies, and imaging changes, whereas our findings suggest that MC-related transcriptomic features may capture microenvironmental activity states that are not readily reflected by conventional clinical indicators, thereby providing complementary information for molecular stratification of RA patients. In the context of rapidly developing precision medicine, if the robustness and generalizability of this model can be further validated in prospective cohorts, it may help identify patient subsets with more active inflammation, more pronounced synovial hyperplasia, and potentially greater sensitivity to specific targeted therapies.

Nevertheless, several limitations of this study should be acknowledged. First, the core findings were mainly derived from retrospective integrative analyses. Although the scRNA-seq discovery cohort comprising 17 RA synovial samples provided a reasonable foundation for identifying major cell states and MC subpopulations, this sample size may still limit our ability to fully capture the diversity of MC subsets across different RA patient demographics, disease stages, and clinical phenotypes. In contrast, the relatively large bulk RNA-seq cohort comprising 218 RA synovial samples represents an important strength of this study, supporting MC-related molecular stratification and downstream validation at a larger sample scale. Nevertheless, potential biases arising from differences in platforms, sample-processing procedures, and clinical backgrounds across datasets cannot be excluded. Second, although we strengthened the identification and biological interpretation of MC subpopulations by examining canonical mast cell markers and integrating marker-gene expression, functional enrichment, transcription factor activity, inferred developmental trajectories, cell-cell communication patterns, and bulk-cohort molecular stratification, these findings remain primarily based on bioinformatic analyses and still lack sufficient experimental validation. Future studies incorporating flow cytometry, spatial transcriptomics, immunofluorescence colocalization, MC-fibroblast or MC-immune cell co-culture systems, and functional perturbation assays will be needed to confirm the actual existence, spatial distribution, developmental relationships, and biological functions of these subpopulations, as well as their direct interactions with macrophages, T cells, fibroblasts, and other immune or stromal cell populations. Third, although virus infection-related pathways were significantly enriched in the high-risk subtype, this finding more likely reflects similarities at the level of transcriptional programs rather than direct evidence that specific pathogens drive subtype divergence in RA. Finally, the clinical value of the diagnostic model remains to be validated in independent, prospective real-world cohorts with complete treatment and outcome information, particularly regarding its ability to predict treatment response, disease activity, and structural damage progression.

## Conclusion

This study revealed substantial heterogeneity of mast cells within the RA synovial microenvironment. Distinct MC subpopulations exhibited different functional states and communication patterns with immune cells, suggesting their potential roles in inflammatory regulation and immune crosstalk. MC-related molecular stratification identified RA subtypes with distinct inflammatory and immune infiltration profiles, among which the C2/high-risk subtype showed stronger inflammatory activation and tissue-destructive features. These findings highlight the importance of MC heterogeneity in RA progression and suggest that MC-associated signatures may provide new insights for patient stratification, biomarker discovery, and therapeutic targeting.

## Data Availability

The HMU scRNA-seq and bulk RNA-seq datasets presented in this article are not publicly available because they were generated from synovial tissue samples of patients with rheumatoid arthritis treated at the Second Affiliated Hospital of Harbin Medical University. These patient-derived individual-level transcriptomic datasets may contain sensitive genetic and clinically relevant information. Public deposition of the raw sequencing data in an open repository may pose potential risks to participant privacy and re-identification and is not covered by the scope of the original informed consent or applicable institutional and national data governance requirements. Requests to access the datasets should be directed to the corresponding author and will be considered in accordance with applicable ethical, institutional, and regulatory requirements.
