# Supplementary material for: Heterogeneity and molecular typing characteristics of mast cells in rheumatoid arthritis
Source: Front Mol Biosci. 2026 Jul 28;13:1898923. doi: 10.3389/fmolb.2026.1898923 (PMC13457048; doi:10.3389/fmolb.2026.1898923)
Supplement: Supplementary file 1 [file DataSheet1.pdf]

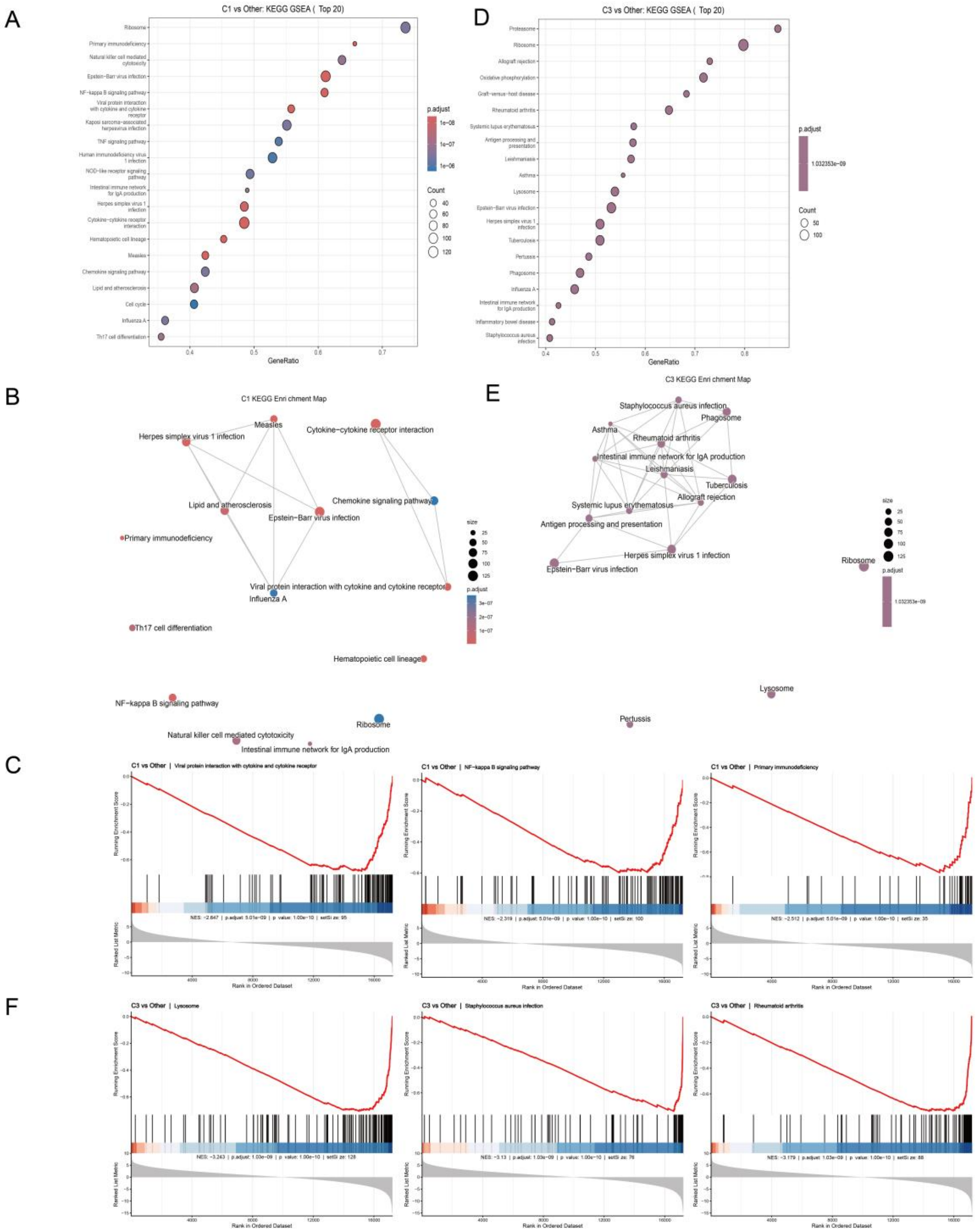

Supplementary Figure 1. KEGG GSEA enrichment analysis of C1 and C3 clusters compared with the other groups. (A, D) Bubble plots showing the top 20 significantly enriched KEGG pathways in the C1 vs. Other and C3 vs. Other comparisons, respectively. (B, E) Enrichment maps for the KEGG pathways in the C1 vs. Other and C3 vs. Other comparisons, respectively. Each node represents a significantly enriched pathway. Node size indicates the gene set size or enriched gene count, node color indicates the significance level, and edges indicate shared genes between pathways. (C, F) Representative GSEA enrichment plots for significantly enriched pathways in

the C1 vs. Other and C3 vs. Other comparisons, respectively. The red curve indicates the running enrichment score, the black vertical bars indicate the positions of pathway genes in the ranked gene list, and the color bar at the bottom indicates the direction of gene ranking correlation.

Supplementary Table 1, Differences in immune cell infiltration between high- and low-risk groups.

Cell_Type	Mean_HighRisk	Mean_LowRisk	Diff	P_Value
Activated CD4 T cell	0.281540248	0.174184097	0.10735615	4.47E-18
Activated dendritic cell	0.440664264	0.351908613	0.088755651	1.40E-17
Monocyte	0.550794756	0.483352498	0.067442258	5.85E-16
Macrophage	0.571861736	0.523543029	0.048318706	6.56E-14
Memory B cell	0.188852317	0.038124151	0.150728166	9.78E-14
MDSC	0.345854796	0.287363814	0.058490982	1.06E-13
Plasmacytoid dendritic cell	0.229373637	0.171428584	0.057945052	5.69E-13
Effector memeory CD8 T cell	0.168273243	-0.011604369	0.179877611	1.52E-12
Gamma delta T cell	0.152987225	-0.004819276	0.157806501	1.52E-12
Effector memeory CD4 T cell	0.129722804	-0.018771637	0.148494441	1.84E-12
Type 1 T helper cell	0.176164539	0.068060877	0.108103662	2.60E-12
Regulatory T cell	0.102077023	0.036545921	0.065531102	7.31E-12
CD56dim natural killer cell	0.083492398	-0.01003094	0.093523339	9.74E-12
Central memory CD8 T cell	0.264564783	0.111457496	0.153107286	1.05E-11
T follicular helper cell	0.128154226	0.022837883	0.105316343	3.71E-11
Natural killer T cell	0.176008052	0.039739428	0.136268624	5.41E-11
Activated CD8 T cell	0.085231053	-0.056153487	0.14138454	6.29E-11
Type 2 T helper cell	0.091534069	0.025357902	0.066176166	8.49E-11
Natural killer cell	-0.160708109	-0.24819679	0.087488681	1.58E-10
Central memory CD4 T cell	0.262468813	0.101499901	0.160968912	5.95E-10
Type 17 T helper cell	0.122307217	0.053927653	0.068379564	1.07E-09
Eosinophil	-0.093393596	-0.164931453	0.071537858	5.24E-09
Activated B cell	0.097389718	-0.067100563	0.164490281	1.42E-08
Neutrophil	0.191891123	0.125078143	0.066812979	3.92E-08
Immature B cell	0.061078981	-0.104479429	0.16555841	6.78E-08
CD56bright natural killer cell	0.281565794	0.228882059	0.052683735	9.31E-07
Immature dendritic cell	0.169174341	0.156133984	0.013040357	0.029249636
Mast cell	0.124171736	0.1549537	-0.03078196	0.220920783
